# Mitral annular calcification severity and invasive hemodynamic confirmation of HFpEF: A prospective observational study

**DOI:** 10.1097/MD.0000000000050835

**Published:** 2026-09-25

**Authors:** Gizem Cabuk, Burcu Uludag Artun

**Affiliations:** a Department of Cardiology, İzmir City Hospital, İzmir, Türkiye.

**Keywords:** calcinosis, diastolic, echocardiography, heart failure, mitral valve, pulmonary wedge pressure

## Abstract

Mitral annular calcification (MAC) is a common degenerative process in older adults that can impair mitral valve compliance, elevate transmitral gradients, and increase left ventricular filling pressure. However, the extent to which increasing MAC severity parallels invasive hemodynamic evidence of heart failure with preserved ejection fraction (HFpEF) and established diagnostic algorithms remains uncertain. This was a single-center cross-sectional study. We evaluated 160 patients referred for HFpEF assessment who underwent comprehensive echocardiography. MAC severity was graded from 1 to 4 (n = 40 per group). HFpEF was defined invasively as a pulmonary capillary wedge pressure of ≥15 mm Hg at rest or ≥18 mm Hg after a standardized fluid challenge. Multivariable regression models tested the independent association between MAC grade (per 1-category increase) and invasive HFpEF diagnosis, as well as continuous H_2_FPEF (Heavy, Hypertensive, Atrial fibrillation, Pulmonary hypertension, Elder, Filling pressure score) and HFA-PEFF (Heart Failure Association Pretest Assessment, Echocardiography and Natriuretic Peptide, Functional Testing, Final Etiology) scores, adjusting for natriuretic peptide levels and key echocardiographic diastolic parameters. Participants were older (mean age, 76 years) and two-thirds were female, with preserved left ventricular ejection fraction (~59%). Higher MAC severity was associated with progressive elevations in pulmonary capillary wedge pressure (13.0–18.0 mm Hg at rest; 16.0–24.5 mm Hg after fluid challenge; *P* < .001) and, among catheterized participants, with a higher proportion meeting invasive HFpEF criteria (28.1%, 30.0%, 60.0%, and 85.0% across grades 1–4, respectively; *P* < .001). MAC grade was independently associated with invasively defined HFpEF (odds ratio, 1.78; 95% confidence interval, 1.22–2.38; *P* = .03), H_2_FPEF (β, 0.31; *P* = .03), and HFA-PEFF (β, 0.42; *P* = .004) scores. N-terminal pro–B-type natriuretic peptide was correlated with all outcomes. MAC severity showed a graded relationship with diastolic burden, invasive HFpEF confirmation, and diagnostic scores, suggesting that advanced MAC is associated with a hemodynamic profile consistent with HFpEF.

## 1. Introduction

Heart failure with preserved ejection fraction (HFpEF) is characterized by a left ventricular ejection fraction (LVEF) ≥ 50%. This condition involves myocardial impairment, which leads to a disproportionately elevated left atrial pressure (LAP) needed to sustain adequate cardiac output.^[1]^ It is believed to be the predominant type of heart failure (HF), with an increasing prevalence compared with that of HF with reduced ejection fraction (HFrEF), largely due to an aging population and an increase in metabolic comorbidities, including systemic hypertension, diabetes mellitus (DM), and obesity. Establishing the diagnosis necessitates the identification of objective indicators of elevated filling pressure, with echocardiography being pivotal in this assessment.^[2]^

Recently, 2 diagnostic algorithms have been introduced to assess the likelihood of HFpEF in patients presenting with dyspnea: the H_2_FPEF (Heavy, Hypertensive, Atrial fibrillation, Pulmonary hypertension, Elder, Filling pressure score) and HFA-PEFF (Heart Failure Association Pretest Assessment, Echocardiography and Natriuretic Peptide, Functional Testing, Final Etiology) scoring systems.^[3,4]^ Both diagnostic tools yield categorical scores to evaluate the probability of HFpEF, with H_2_FPEF scores ranging from 0 to 9 and HFA-PEFF scores ranging from 0 to 6. For patients in the intermediate-probability range, both algorithms recommend additional functional or invasive assessment to clarify the diagnosis.

Mitral annular calcification (MAC) is the deposition of calcium within the fibrous structure of the mitral valve. In most instances, it follows an asymptomatic trajectory and is frequently discovered incidentally during echocardiographic examination. The incidence of MAC has been observed to increase, correlating with an increasing number of elderly patients who often present with multiple comorbidities such as chronic kidney disease (CKD), hypertension, and DM.^[5,6]^

Emerging research from multiple studies supports a connection between MAC and HFpEF. MAC has been associated with HF and adverse cardiac remodeling; however, the observational literature does not establish a causal role.^[7]^ The severity and presence of MAC correlate with compromised left ventricular (LV) systolic and diastolic functions; however, caution is advised when interpreting diastolic parameters in individuals with MAC, as annular calcification can affect e′ measurement, which is an indicator of myocardial relaxation.^[8,9]^ Additionally, mitral E-wave velocity tends to increase as mitral calcification worsens, primarily due to relative stenosis of the mitral inflow.^[8,9]^

A grading system for MAC has recently been introduced, which categorizes MAC based on parasternal short-axis echocardiographic images. MAC is divided into 4 grades, with grade 1 representing the least and grade 4 representing the most extensive calcification.^[5]^ Because MAC severity and HFpEF probability share several age-related and cardiometabolic correlates, including hypertension and DM, we aimed to evaluate the relationship between MAC severity, established HFpEF diagnostic scores, and invasive hemodynamic evidence of HFpEF.

## 2. Materials and methods

### 2.1. Study design

This was a single-center, prospective, observational study with prospective enrollment using predefined MAC-grade strata. This study had a cross-sectional design, and participants were evaluated at baseline without longitudinal follow-up for prognostic outcomes. All procedures conformed to the Declaration of Helsinki and were approved by the Institutional Ethics Committee of İzmir City Hospital (protocol code 2024/178; approval date: November 6, 2024). Written informed consent was obtained from all participants prior to inclusion in the study.

The required sample size was determined a priori to ensure adequate statistical power to detect clinically meaningful associations between MAC grade and diagnostic endpoints related to HFpEF. Based on preliminary data and prior literature, a minimum of 160 participants were estimated to provide 80% statistical power to detect a moderate effect size (Cohen’s *f*^2^ = 0.15) at a two-sided α level of 0.05. The computation was performed using the G*Power software (version 3.1.9.7, University of Düsseldorf, Germany).

### 2.2. Study population

An initial screening cohort of 225 MAC-positive individuals was prospectively assembled. Eligible patients were enrolled using predefined MAC-grade strata (grades 1–4; target n = 40 per group) to ensure balanced representation across severity categories for internal comparisons. Enrollment within each stratum continued until the prespecified target was reached. Investigators responsible for group allocation were blinded to clinical, laboratory, and echocardiographic variables other than MAC grade. This sampling strategy was designed for internal comparisons and does not reflect the natural distribution of MAC severity in an unselected clinical population.

The exclusion criteria were the presence of HF with a reduced or mildly reduced ejection fraction (HFrEF or heart failure with mildly reduced ejection fraction), moderate-to-severe valvular heart disease, prior cardiac surgery (including valve replacement or repair), myocardial infarction, active myocarditis or pericarditis, systemic inflammatory disorders, pregnancy, WHO group 1 pulmonary hypertension, advanced CKD (stage 4–5), cardiac infiltrative or storage diseases (amyloidosis, sarcoidosis), hypertrophic or restrictive cardiomyopathy, and constrictive pericarditis.

This stratified enrollment strategy was intentionally designed to ensure balanced representation across MAC grades for internal comparison; however, it does not reflect the real-world distribution of MAC severity and may limit external generalizability.

### 2.3. Two-/three-dimensional echocardiography

All echocardiographic examinations were performed using a GE Vivid E95 ultrasound system (GE Healthcare, Chicago) equipped with an M5Sc-D sector-array transducer. Comprehensive 2-dimensional Doppler and tissue Doppler measurements were obtained in accordance with the recommendations of the American Society of Echocardiography (ASE).^[10]^

MAC was defined as an echo-dense, shelf-like structure with an irregular, lumpy texture encircling the mitral annulus and producing posterior acoustic shadowing, typically visualized in parasternal long- and short-axis views. The severity of MAC was graded qualitatively from 1 to 4 based on the circumferential extent of calcification at the mitral annulus: grade 1, <90° and noncontiguous; grade 2, 90° to <180°; grade 3, 180° to <270°; grade 4, 270° to circumferential involvement.^[5]^

The following echocardiographic parameters were assessed as estimates of the left ventricular filling pressure: transmitral flow pattern, left atrial volume index (LAVI), E/A ratio (ratio of early [E] to late [A] mitral inflow velocity), ratio of early diastolic transmitral inflow velocity to average mitral e′ velocity (E/e′), isovolumic relaxation time (IVRT), tricuspid regurgitation velocity (TRV), left ventricular mass index, and relative wall thickness.

LAVI was quantified using 3-dimensional echocardiography by tracing the endocardial borders at end-systole from apical 4-chamber and 2-chamber views, followed by volumetric reconstruction using dedicated vendor software. This approach minimizes geometric assumptions and improves the accuracy of atrial volume assessment compared with conventional biplane methods.

Because significant MAC can artifactually elevate E/e′ by reducing annular excursion, diastolic evaluation followed the ASE 2025 special-population algorithm: E/A <0.8 indicates normal LAP; E/A > 1.8 indicates elevated LAP; and when E/A was 0.8 to 1.8, IVRT ≥80 ms suggested normal and IVRT <80 ms elevated LAP. This strategy is consistent with prior evidence that validates E/A and IVRT in MAC.^[11]^

To evaluate the measurement reproducibility, all echocardiographic analyses were independently performed by 2 experienced operators who were blinded to each other’s results. Interobserver variability was assessed for key quantitative and qualitative parameters, including MAC grade, LAVI, E/A ratio, E/e′ ratio, IVRT, LVEF, and global longitudinal strain (GLS). Agreement between observers was quantified using the intraclass correlation coefficient with a 2-way random-effects model and absolute agreement definition. Reproducibility analysis demonstrated excellent interobserver reliability (intraclass correlation coefficient = 0.92; 95% confidence interval [CI], 0.88–0.95; *P* < .001), supporting the robustness and internal consistency of the echocardiographic dataset.

### 2.4. Speckle-tracking echocardiography and global longitudinal strain calculation

GLS was assessed using speckle-tracking echocardiography. Standard echocardiographic images were obtained in the apical 4-, 2-, and 3-chamber views, ensuring optimal visualization of the left ventricle (LV). The images were acquired at a frame rate of at least 60 frames per second to enhance tracking accuracy. Using dedicated software, the endocardial border of the LV was manually traced in each view, allowing the software to automatically track the myocardial motion throughout the cardiac cycle. GLS was calculated as the average strain value from the 3 apical views and expressed as a percentage of the total myocardial length change during systole. A GLS value of <−16% was considered indicative of impaired LV systolic function.^[12]^

### 2.5. *H*_*2*_*FPEF and HFA-PEFF scores*

HFA-PEFF (Heart Failure Association of ESC diagnostic algorithm: P [Pretest Assessment], E [Echocardiographic and Natriuretic Peptide score], F1 [Functional testing in Case of Uncertainty], F2 [Final Aetiology]); H_2_FPEF (Heavy [BMI >30 kg/m^2^], hypertensive [use of ≥2 antihypertensive medications], atrial fibrillation [paroxysmal or persistent], pulmonary hypertension [Doppler echocardiographic estimated pulmonary artery systolic pressure >35 mm Hg], elderly [age >60 years], and filling pressure [Doppler echocardiographic E/e′ >9]). The H_2_FPEF and HFA-PEFF scores were calculated according to previously published algorithms.^[3,4]^

### 2.6. Right heart catheterization

All patients initially underwent comprehensive echocardiographic assessments, and both H_2_FPEF and HFA-PEFF scores were calculated. In accordance with the current guideline recommendations, patients with intermediate- or high-probability zone scores for both algorithms (H_2_FPEF ≥2 and HFA-PEFF ≥2, n = 152) were referred for invasive hemodynamic evaluation with right heart catheterization (RHC). In these patients, baseline pressures were measured at rest, followed by a rapid fluid challenge to unmask latent elevations in the filling pressures. Conversely, individuals with low scores (0 or 1 in both scoring systems, n = 8) were considered unlikely to have HFpEF and therefore were not subjected to invasive assessment. This stepwise approach ensured that catheterization was reserved for cases in which noninvasive diagnostic scores were inconclusive or strongly suggestive of HFpEF. RHC was performed via the femoral or internal jugular vein, using standard techniques. The pressures recorded included right atrial pressure, pulmonary artery pressure, and pulmonary capillary wedge pressure (PCWP), all measured at end-expiration.

For the fluid challenge, 500 mL isotonic saline (0.9% NaCl) was infused intravenously over 5 to 10 minutes. Hemodynamic parameters were reassessed immediately after the infusion. An increase in PCWP to ≥18 mm Hg was considered abnormal in accordance with published consensus recommendations.^[13]^

PCWP ≥15 mm Hg at rest or ≥18 mm Hg following a standardized fluid challenge was defined as meeting the invasive hemodynamic criteria for HFpEF in accordance with current consensus recommendations.^[13]^

All RHC procedures were performed by experienced interventional cardiologists in a dedicated cardiac catheterization laboratory under sterile conditions, with continuous monitoring of the heart rate, systemic blood pressure, and oxygen saturation throughout the procedure.

### 2.7. Statistical analysis

All statistical analyses were prespecified in a reproducible analysis plan prior to database lock and were conducted using R software (version 4.3.1; R Foundation for Statistical Computing). Invasive HFpEF regression analyses were performed in the subset of patients who underwent RHC (n = 152). Continuous score analyses included the full cohort (n = 160). Continuous variables were evaluated for outliers and normality using visual inspection and the Shapiro–Wilk test; skewed variables (e.g., N-terminal pro–B-type natriuretic peptide [NT-proBNP]) were log-transformed to normalize the distributions. Categorical variables were presented as counts and percentages, and continuous variables as mean ± standard deviation or median (interquartile range), as appropriate. The primary endpoint was invasively confirmed HFpEF defined by PCWP criteria. Secondary endpoints included continuous H_2_FPEF and HFA-PEFF scores.

Between-group comparisons across the 4 MAC grades (n = 40 each) were performed using one-way analysis of variance or Kruskal–Wallis tests for continuous variables and χ^2^ or Fisher’s exact tests for categorical variables. Global *P* values <.05 were followed by pairwise post hoc tests with Holm–Bonferroni correction to control the family-wise error rate.

Three prespecified multivariable regression models were constructed to examine the independent association between MAC grade (exposure variable) and diagnostic endpoints: the presence of invasively defined HFpEF (binary logistic regression), continuous H_2_FPEF score, and continuous HFA-PEFF score (both multiple linear regressions). Covariates were selected a priori, based on physiological plausibility and previous studies. All variables were entered simultaneously, without stepwise procedures, to avoid data-driven bias. Continuous predictors were standardized (*z*-scores) to facilitate the comparison of effect sizes.

Model assumptions and multicollinearity were assessed using standard diagnostic procedures. Logistic-model discrimination and calibration and linear-model residual diagnostics were evaluated before interpretation of the final models. Results are reported as odds ratios (ORs) with 95% CIs for logistic regression and standardized β coefficients with 95% CIs for linear regression. Statistical significance was defined as a two-sided *P* < .05; CIs were calculated using robust (Huber-White) standard errors.

### 2.8. Data availability statement

All analysis scripts, the data dictionary, and code for model replication are not publicly available but are available from the corresponding author upon reasonable request, ensuring full reproducibility of the statistical workflow. Data were stored in controlled conditions at İzmir City Hospital, University of Health Sciences Institute.

## 3. Results

The mean age of the cohort was 76 ± 8 years, and 67% were women. The mean body mass index (BMI) was 29.3 kg/m^2^, and the LVEF was approximately 59%, consistent with preserved systolic function. Across MAC grades, there were no significant differences in age, sex distribution, BMI, hypertension, DM, or atrial fibrillation (AF) prevalence (all *P* > .10), reflecting the balanced group composition intentionally achieved during patient selection using the structured, blinded enrollment algorithm described in the Methods section (Table 1). Symptom burden increased with MAC severity; New York Heart Association class increased from ~1.4 in grades 1 and 2 to 2.1 in grade 3 and 2.5 in grade 4 (overall *P* = .001). GLS showed progressive impairment, from approximately −20% in MAC grade 1 to −16% in MAC grade 4 (*P* = .001), whereas LVEF remained similar across the groups (overall *P* = .48; Table 1).

**Table 1:** Baseline characteristics, echocardiographic measurements, and laboratory results of the study groups.

Parameter	MAC grade 1 (n = 40)	MAC grade 2 (n = 40)	MAC grade 3 (n = 40)	MAC grade 4 (n = 40)	Pairwise *P* values
Age, yr	76.8 ± 9.1	75.1 ± 7.0	75.4 ± 7.5	77.0 ± 6.6	p_1vs2_ = .34
					p_1vs3_ = .43
					p_1vs4_ = .92
					p_2vs3_ = .86
					p_2vs4_ = .21
					p_3vs4_ = .30
Sex, female	27 (67.5)	27 (67.5)	30 (75.0)	23 (57.5)	p_1vs2_ > .99
					p_1vs3_ = .62
					p_1vs4_ = .48
					p_2vs3_ = .62
					p_2vs4_ = .48
					p_3vs4_ = .15
Hypertension	37 (92.5)	35 (87.5)	40 (100.0)	38 (95.0)	p_1vs2_ = .71
					p_1vs3_ = .24
					p_1vs4_ > .99
					p_2vs3_ = .05
					p_2vs4_ = .43
					p_3vs4_ = .49
Diabetes mellitus	14 (35.0)	14 (35.0)	18 (45.0)	21 (52.5)	p_1vs2_ > .99
					p_1vs3_ = .49
					p_1vs4_ = .17
					p_2vs3_ = .49
					p_2vs4_ = .17
					p_3vs4_ = .65
Dyslipidemia	25 (62.5)	21 (52.5)	18 (45.0)	27 (67.5)	p_1vs2_ = .49
					p_1vs3_ = .17
					p_1vs4_ = .81
					p_2vs3_ = .65
					p_2vs4_ = .25
					p_3vs4_ = .07
Smoking	12 (30.0%)	8 (20.0%)	3 (7.5%)	14 (35.0%)	p_1vs2_ = .43
					p_1vs3_ = .02
					p_1vs4_ = .81
					p_2vs3_ = .19
					p_2vs4_ = .21
					p_3vs4_ = .005
CAD history	6 (15.0)	13 (32.5)	15 (37.5)	20 (50.0)	p_1vs2_ = .11
					p_1vs3_ = .04
					p_1vs4_ = .002
					p_2vs3_ = .81
					p_2vs4_ = .17
					p_3vs4_ = .36
AF	8 (20.0)	10 (25.0)	12 (30.0)	17 (42.5)	p_1vs2_ = .79
					p_1vs3_ = .43
					p_1vs4_ = .05
					p_2vs3_ = .80
					p_2vs4_ = .15
					p_3vs4_ = .35
BMI, kg/m^2^	28.2 ± 6.5	28.9 ± 6.3	30.1 ± 4.1	30.8 ± 3.1	p_1vs2_ = .06
					p_1vs3_ = .05
					p_1vs4_ = .05
					p_2vs3_ = .42
					p_2vs4_ = .26
					p_3vs4_ = .71
NYHA class	1.4 ± 0.6	1.4 ± 0.7	2.1 ± 0.7	2.5 ± 0.7	p_1vs2_ = .56
					p_1vs3_ < .001
					p_1vs4_ < .001
					p_2vs3_ < .001
					p_2vs4_ < .001
					p_3vs4_ = .04
LVEF, %	60.1 ± 4.4	59.0 ± 4.1	59.2 ± 4.5	58.1 ± 3.9	p_1vs2_ = .69
					p_1vs3_ = .96
					p_1vs4_ = .15
					p_2vs3_ = .73
					p_2vs4_ = .29
					p_3vs4_ = .18
GLS, −%	−20.2 ± 3.6	−19.5 ± 3.3	−19.0 ± 3.0	−16.0 ± 3.0	p_1vs2_ = .32
					p_1vs3_ = .11
					p_1vs4_ < .001
					p_2vs3_ = .54
					p_2vs4_ < .001
					p_3vs4_ < .001
RWT*	0.42 ± 0.06	0.45 ± 0.08	0.48 ± 0.08	0.47 ± 0.05	p_1vs2_ = .09
					p_1vs3_ < .001
					p_1vs4_ < .001
					p_2vs3_ = .06
					p_2vs4_ = .11
					p_3vs4_ = .39
LVMI, g/m^2^	84.2 ± 19.9	91.0 ± 21.2	94.3 ± 21.1	110.7 ± 9.8	p_1vs2_ = .14
					p_1vs3_ = .03
					p_1vs4_ < .001
					p_2vs3_ = .49
					p_2vs4_ < .001
					p_3vs4_ < .001
LAVI, mL/m^2^	26.2 ± 8.0	36.1 ± 10.7	38.1 ± 6.2	39.9 ± 5.0	p_1vs2_ < .001
					p_1vs3_ < .001
					p_1vs4_ < .001
					p_2vs3_ = .30
					p_2vs4_ = .04
					p_3vs4_ = .15
TR velocity, m/s	1.9 ± 0.4	2.4 ± 0.5	2.6 ± 0.6	3.3 ± 0.3	p_1vs2_ < .001
					p_1vs3_ < .001
					p_1vs4_ < .001
					p_2vs3_ = .06
					p_2vs4_ < .001
					p_3vs4_ < .001
E/e′	8.9 ± 2.6	13.2 ± 4.9	13.7 ± 3.4	14.2 ± 4.3	p_1vs2_ < .001
					p_1vs3_ < .001
					p_1vs4_ < .001
					p_2vs3_ = .25
					p_2vs4_ = .06
					p_3vs4_ = .17
E/A	1.1 ± 0.2	1.1 ± 0.2	1.3 ± 0.2	1.4 ± 0.2	p_1vs2_ = .04
					p_1vs3_ < .001
					p_1vs4_ < .001
					p_2vs3_ = .005
					p_2vs4_ < .001
					p_3vs4_ = .01
IVRT, ms	112.0 ± 6.4	105.7 ± 8.3	98.6 ± 11.9	92.6 ± 14.9	p_1vs2_ < .001
					p_1vs3_ < .001
					p_1vs4_ < .001
					p_2vs3_ = .02
					p_2vs4_ < .001
					p_3vs4_ = .008
NT-proBNP, pg/mL	183.9 ± 86.0	572.8 ± 445.7	773.7 ± 488.0	1742.8 ± 945.4	p_1vs2_ < .001
					p_1vs3_ < .001
					p_1vs4_ < .001
					p_2vs3_ = .05
					p_2vs4_ < .001
					p_3vs4_ < .001
Glucose, mg/dL	98.6 ± 19.6	98.3 ± 14.2	104.3 ± 21.0	103.8 ± 26.2	p_1vs2_ = .93
					p_1vs3_ = .21
					p_1vs4_ = .32
					p_2vs3_ = .14
					p_2vs4_ = .25
					p_3vs4_ = .92
Creatinine, mg/dL	0.9 ± 0.1	1.0 ± 0.3	0.9 ± 0.2	1.0 ± 0.2	p_1vs2_ = .41
					p_1vs3_ = .24
					p_1vs4_ = .08
					p_2vs3_ = .11
					p_2vs4_ = .55
					p_3vs4_ = .01
AST, U/L	17.8 ± 4.5	18.7 ± 6.1	18.7 ± 3.9	20.0 ± 4.1	p_1vs2_ = .41
					p_1vs3_ = .30
					p_1vs4_ = .02
					p_2vs3_ > .99
					p_2vs4_ = .27
					p_3vs4_ = .15
ALT, U/L	15.4 ± 5.2	16.8 ± 7.5	15.6 ± 5.3	18.1 ± 4.5	p_1vs2_ = .71
					p_1vs3_ = .93
					p_1vs4_ = .04
					p_2vs3_ = .70
					p_2vs4_ = .11
					p_3vs4_ = .02
Hemoglobin, g/dL	13.0 ± 1.4	13.0 ± 1.5	13.0 ± 1.7	13.1 ± 1.3	p_1vs2_ = .98
					p_1vs3_ = .82
					p_1vs4_ = .69
					p_2vs3_ = .83
					p_2vs4_ = .71
					p_3vs4_ = .89
WBC, ×10^3^/µL	7797.5 ± 1316.6	7015.9 ± 1524.4	7104.3 ± 2244.6	7483.4 ± 1654.6	p_1vs2_ = .01
					p_1vs3_ = .09
					p_1vs4_ = .35
					p_2vs3_ = .83
					p_2vs4_ = .19
					p_3vs4_ = .39
Platelet, ×10^3^/µL	259.4 ± 78.2	241.1 ± 40.7	239.7 ± 78.9	298.7 ± 75.6	p_1vs2_ = .19
					p_1vs3_ = .26
					p_1vs4_ = .02
					p_2vs3_ = .91
					p_2vs4_ = .001
					p_3vs4_ = .001
LDL, mg/dL	120.5 ± 49.4	138.1 ± 40.5	133.3 ± 40.3	132.3 ± 28.1	p_1vs2_ = .08
					p_1vs3_ = .20
					p_1vs4_ = .19
					p_2vs3_ = .60
					p_2vs4_ = .45
					p_3vs4_ = .89
HDL, mg/dL	46.9 ± 13.1	49.1 ± 11.3	43.3 ± 13.1	41.4 ± 9.3	p_1vs2_ = .26
					p_1vs3_ = .51
					p_1vs4_ = .04
					p_2vs3_ = .07
					p_2vs4_ = .001
					p_3vs4_ = .05
Triglyceride, mg/dL	146.3 ± 58.4	143.7 ± 71.7	181.1 ± 59.5	171.9 ± 51.9	p_1vs2_ = .56
					p_1vs3_ = .01
					p_1vs4_ = .05
					p_2vs3_ = .003
					p_2vs4_ = .01
					p_3vs4_ = .39
Total cholesterol, mg/dL	195.0 ± 40.8	195.2 ± 34.8	197.3 ± 29.1	199.3 ± 39.5	p_1vs2_ = .88
					p_1vs3_ = .61
					p_1vs4_ = .63
					p_2vs3_ = .35
					p_2vs4_ = .41
					p_3vs4_ = .86

AF = atrial fibrillation, ALT = alanine aminotransferase, AST = aspartate aminotransferase, BMI = body mass index, CAD = coronary artery disease, E/A = ratio of early (E) to late (A) mitral inflow velocity, GLS = global longitudinal strain, HDL = high-density lipoprotein, IVRT = isovolumic relaxation time, LAVI = left atrial volume index, LDL = low-density lipoprotein, LVEF = left ventricular ejection fraction, LVIDd = left ventricular internal diameter at end-diastole, LVMI = left ventricular mass index, MAC = mitral annular calcification, NT-proBNP = N-terminal pro–B-type natriuretic peptide, NYHA = New York Heart Association, PWT = posterior wall thickness, RWT = relative wall thickness, TR = tricuspid regurgitation, WBC = white blood cell count.

* RWT = (2 × PWT)/LVIDd.

The PCWP values demonstrated a clear stepwise increase in MAC severity. At rest, the median PCWP increased from 13 (11–14) mm Hg in MAC grade 1 to 18 (17–20) mm Hg in MAC grade 4 (overall *P* < .001; multiple pairwise contrasts significant vs MAC grade 4). Following fluid challenge, PCWP increased from 16 (15–19) mm Hg in MAC grade 1 to 24.5 (20.8–27.0) mm Hg in MAC grade 4 (overall *P* < .001). Correspondingly, the proportion meeting the invasive HFpEF criteria increased from 28.1% (MAC grade 1) and 30.0% (MAC grade 2) to 60% (MAC grade 3) and 85% (MAC grade 4; overall *P* < .001; for pairwise comparisons, see Table 2).

**Table 2:** Invasive PCWP measurements and HFpEF diagnosis of the study groups.

Parameter	MAC grade 1 (n = 32)*	MAC grade 2 (n = 40)	MAC grade 3 (n = 40)	MAC grade 4 (n = 40)	Pairwise *P* values
PCWP at rest, mm Hg	13.0 (11.0–14.0)	14.0 (12.0–14.0)	14.0 (14.0–18.0)	18.0 (17.0–20.0)	p_1vs2_ = .19
					p_1vs3_ < .001
					p_1vs4_ < .001
					p_2vs3_ = .02
					p_2vs4_ < .001
					p_3vs4_ = .002
PCWP after fluid, mm Hg	16.0 (15.8–17.0)	17.0 (16.0–17.0)	17.0 (17.0–22.2)	24.5 (20.8–27.0)	p_1vs2_ = .83
					p_1vs3_ = .001
					p_1vs4_ < .001
					p_2vs3_ = .03
					p_2vs4_ < .001
					p_3vs4_ < .001
HFpEF invasive diagnosis	9 (28.1%)	12 (30.0%)	24 (60.0%)	34 (85.0%)	p_1vs2_ > .99
					p_1vs3_ = .008
					p_1vs4_ < .001
					p_2vs3_ = .07
					p_2vs4_ < .001
					p_3vs4_ = .13

HFpEF = heart failure with preserved ejection fraction, MAC = mitral annular calcification, PCWP = pulmonary capillary wedge pressure.

* Because 8 low-likelihood grade 1 patients did not undergo invasive catheterization, the sample size for grade 1 was n = 32.

Diagnostic scoring systems mirrored these hemodynamic patterns. Median H_2_FPEF scores rose from 2.0 (1.75–3.0) in MAC grade 1 to 6.0 (5–8.25) in MAC grade 4 (*P* < .001), and HFA-PEFF scores rose from 2.0 (1–4) to 5.75 (5.50–6; *P* < .001), with significant pairwise differences between MAC grade 4 and lower grades. In addition to the overall stepwise increase in the median scores, subgroup analyses revealed that patients exceeding specific diagnostic score thresholds exhibited significantly higher MAC grades. This finding reinforces the graded association between increasing MAC severity and the likelihood of HFpEF, as reflected by both diagnostic algorithms (Table 3).

**Table 3:** HFpEF score comparisons of the study groups.

Parameter	MAC grade 1 (n = 40)	MAC grade 2 (n = 40)	MAC grade 3 (n = 40)	MAC grade 4 (n = 40)	Pairwise *P* values
H_2_FPEF score ≥ 6	0 (0.0%)	14 (35.0%)	23 (57.5%)	29 (72.5%)	p_1vs2_ < .001
					p_1vs3_ < .001
					p_1vs4_ < .001
					p_2vs3_ = .43
					p_2vs4_ = .009
					p_3vs4_ > .99
HFA-PEFF score ≥ 5	7 (17.5%)	22 (55.0%)	28 (70.0%)	35 (87.5%)	p_1vs2_ = .006
					p_1vs3_ < .001
					p_1vs4_ < .001
					p_2vs3_ > .99
					p_2vs4_ = .01
					p_3vs4_ = .59
H_2_FPEF score	2.0 (1.75–3.0)	5.0 (2.75–6.0)	6.0 (4.5–6.0)	6.0 (5.0–8.25)	p_1vs2_ < .001
					p_1vs3_ < .001
					p_1vs4_ < .001
					p_2vs3_ = .09
					p_2vs4_ < .001
					p_3vs4_ = .09
HFA-PEFF score	2.0 (1–4)	5.0 (2.75–6.0)	5.0 (4–6)	5.75 (5.5–6)	p_1vs2_ < .001
					p_1vs3_ < .001
					p_1vs4_ < .001
					p_2vs3_ = .10
					p_2vs4_ < .001
					p_3vs4_ < .001

HFA-PEFF (Heart Failure Association of ESC diagnostic algorithm: P [Pretest Assessment], E [Echocardiographic and Natriuretic Peptide score], F1 [Functional testing in Case of Uncertainty], F2 [Final Aetiology]); H_2_FPEF (Heavy [BMI >30 kg/m^2^], hypertensive [use of ≥2 antihypertensive medications], atrial fibrillation [paroxysmal or persistent], pulmonary hypertension [Doppler echocardiographic estimated pulmonary artery systolic pressure >35 mm Hg], Elderly [age >60 years], and filling pressure [Doppler echocardiographic E/e′ >9]).

HFpEF = heart failure with preserved ejection fraction, MAC = mitral annular calcification.

In the binary logistic regression model for invasive HFpEF diagnosis, MAC grade was independently associated with invasively defined HFpEF with an OR of 1.78 (95% CI, 1.22–2.38; *P* = .03) per 1-category increase. NT-proBNP (log) was also independently associated (OR, 2.74; 95% CI, 1.10–6.85; *P* = .03). The other diastolic indices did not reach significance after adjustment (E/A, *P* = .33; LAVI, *P* = .61; GLS *P* = .33; IVRT, *P* = .24; TRV, *P* = .34; Table 4). Figure 1 shows a forest plot of the adjusted ORs with 95% CIs.

**Table 4:** Binary logistic regression results – HFpEF diagnosis.

Variable	OR	CI lower	CI upper	*P* value
MAC grade	1.78	1.22	2.38	.03
NT-proBNP (log)	2.74	1.1	6.85	.03
E/A	1.18	0.84	1.66	.33
LAVI	1.0	0.96	1.04	.61
GLS	0.91	0.76	1.09	.32
IVRT	0.99	0.97	1.01	.24
TR velocity	1.15	0.85	1.56	.34

CI = confidence interval, E/A = ratio of early (E) to late (A) mitral inflow velocity, GLS = global longitudinal strain, HFpEF = heart failure with preserved ejection fraction, IVRT = isovolumic relaxation time, LAVI = left atrial volume index, MAC = mitral annular calcification, NT-proBNP = N-terminal pro–B-type natriuretic peptide, OR = odds ratio, TR = tricuspid regurgitation.

**Figure 1.**
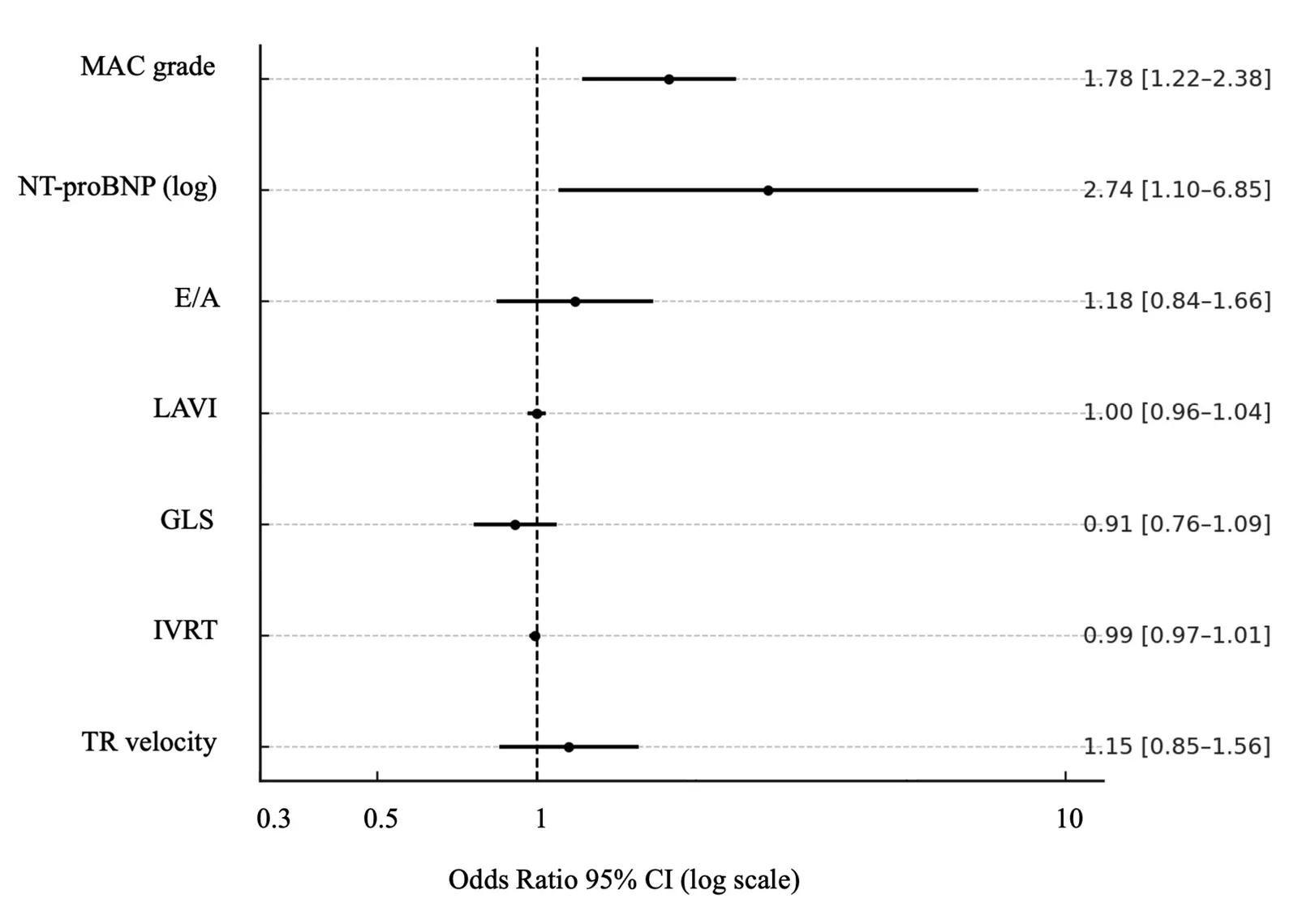
Forest plot demonstrating independent predictors of invasive HFpEF diagnosis in binary logistic regression analysis. CI = confidence interval, GLS = global longitudinal strain, HFpEF = heart failure with preserved ejection fraction, IVRT = isovolumic relaxation time, LAVI = left atrial volume index, MAC = mitral annular calcification, NT-proBNP = N-terminal pro–B-type natriuretic peptide, TR = tricuspid regurgitation.

In the linear regression for H_2_FPEF (continuous), MAC grade was independently associated with higher scores (β, 0.31; 95% CI, 0.03–0.59; *P* = .03), similar to NT-proBNP (log) (β, 0.26; 95% CI, 0.05–0.47; *P* = .01). LAVI and IVRT tended toward association (*P* = .08 and *P* = .09, respectively) but did not meet the significance threshold (Table 5; Fig. 2 shows the forest plot of adjusted β coefficients).

**Table 5:** Multivariable linear regression results – H_2_FPEF score.

Variable	β	CI lower	CI upper	*P* value
MAC grade	0.31	0.03	0.59	.03
NT-proBNP (log)	0.26	0.05	0.47	.01
E/A	0.11	−0.12	0.34	.12
LAVI	0.08	−0.01	0.17	.08
IVRT	−0.07	−0.16	0.01	.09
GLS	−0.12	−0.25	0.01	.05
TR velocity	0.07	−0.09	0.23	.28

CI = confidence interval, E/A = ratio of early (E) to late (A) mitral inflow velocity, GLS = global longitudinal strain, H_2_FPEF = Heavy, Hypertensive, Atrial fibrillation, Pulmonary hypertension, Elder, Filling pressure score, IVRT = isovolumic relaxation time, LAVI = left atrial volume index, MAC = mitral annular calcification, NT-proBNP = N-terminal pro–B-type natriuretic peptide, TR = tricuspid regurgitation.

**Figure 2.**
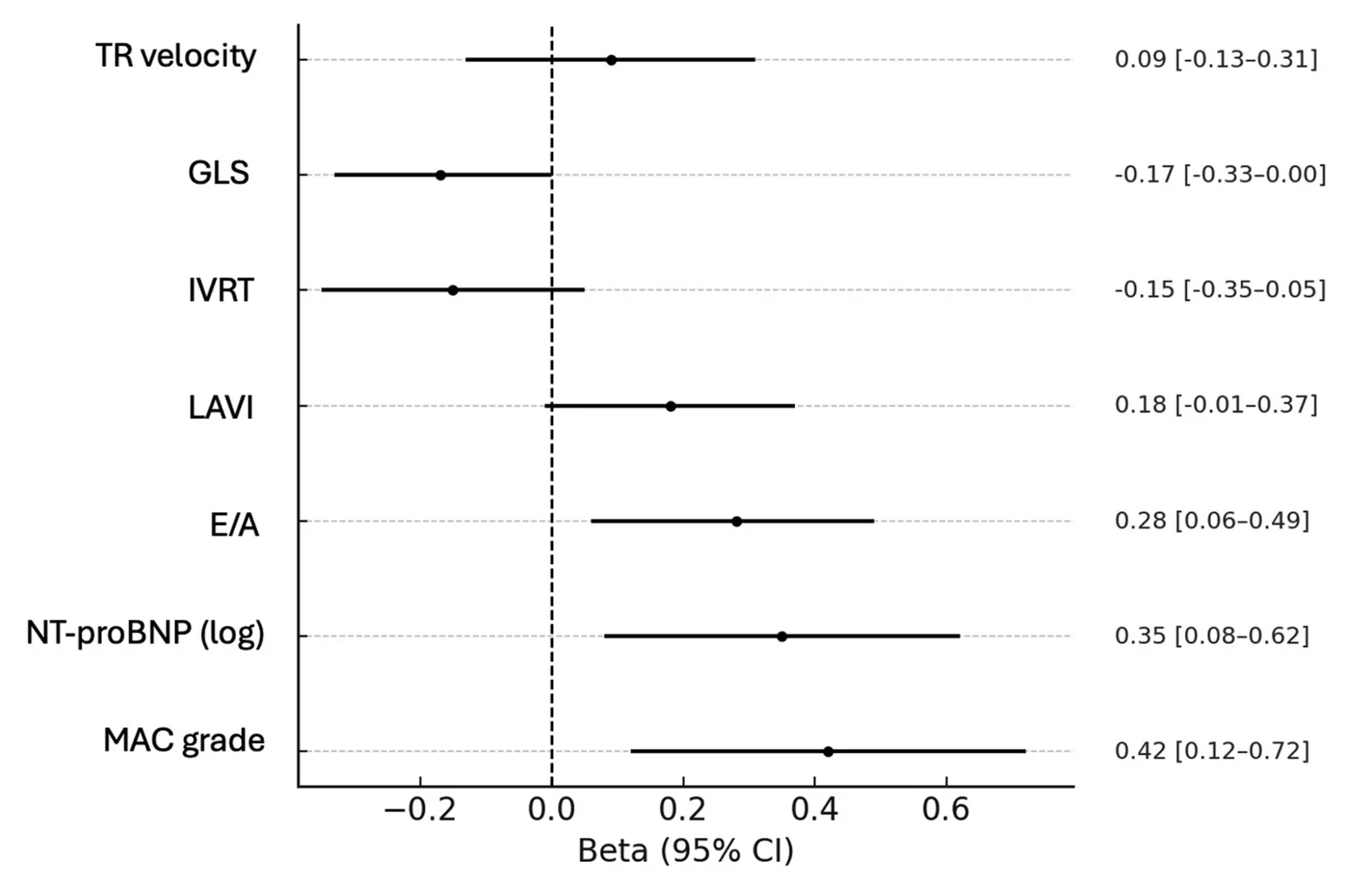
Forest plot of independent predictors of the H_2_FPEF score. CI = confidence interval, GLS = global longitudinal strain, H_2_FPEF = Heavy, Hypertensive, Atrial fibrillation, Pulmonary hypertension, Elder, Filling pressure score, IVRT = isovolumic relaxation time, LAVI = left atrial volume index, MAC = mitral annular calcification, NT-proBNP = N-terminal pro–B-type natriuretic peptide, TR = tricuspid regurgitation.

For HFA-PEFF (continuous), the independent association with MAC grade was stronger (β, 0.42; 95% CI, 0.12–0.72; *P* = .004). NT-proBNP (log) (β, 0.35; 95% CI, 0.08–0.62; *P* = .01) and E/A (β, 0.28; 95% CI, 0.06–0.49; *P* = .02) were also independently related to higher HFA-PEFF scores, whereas LAVI showed a borderline association (*P* = .05; Table 6; Fig. 3 shows the forest plot of adjusted β coefficients).

**Table 6:** Multivariable linear regression results – HFA-PEFF score.

Variable	β	CI lower	CI upper	*P* value
MAC grade	0.42	0.12	0.72	.004
NT-proBNP (log)	0.35	0.08	0.62	.01
E/A	0.28	0.06	0.49	.02
LAVI	0.18	−0.01	0.37	.05
IVRT	−0.15	−0.35	0.05	.07
GLS	−0.17	−0.33	0.0	.05
TR velocity	0.09	−0.13	0.31	.22

CI = confidence interval, E/A = ratio of early (E) to late (A) mitral inflow velocity, GLS = global longitudinal strain, HFA-PEFF = Heart Failure Association Pretest Assessment, Echocardiography and Natriuretic Peptide, Functional Testing, Final Etiology, IVRT = isovolumic relaxation time, LAVI = left atrial volume index, MAC = mitral annular calcification, NT-proBNP = N-terminal pro–B-type natriuretic peptide, TR = tricuspid regurgitation.

**Figure 3.**
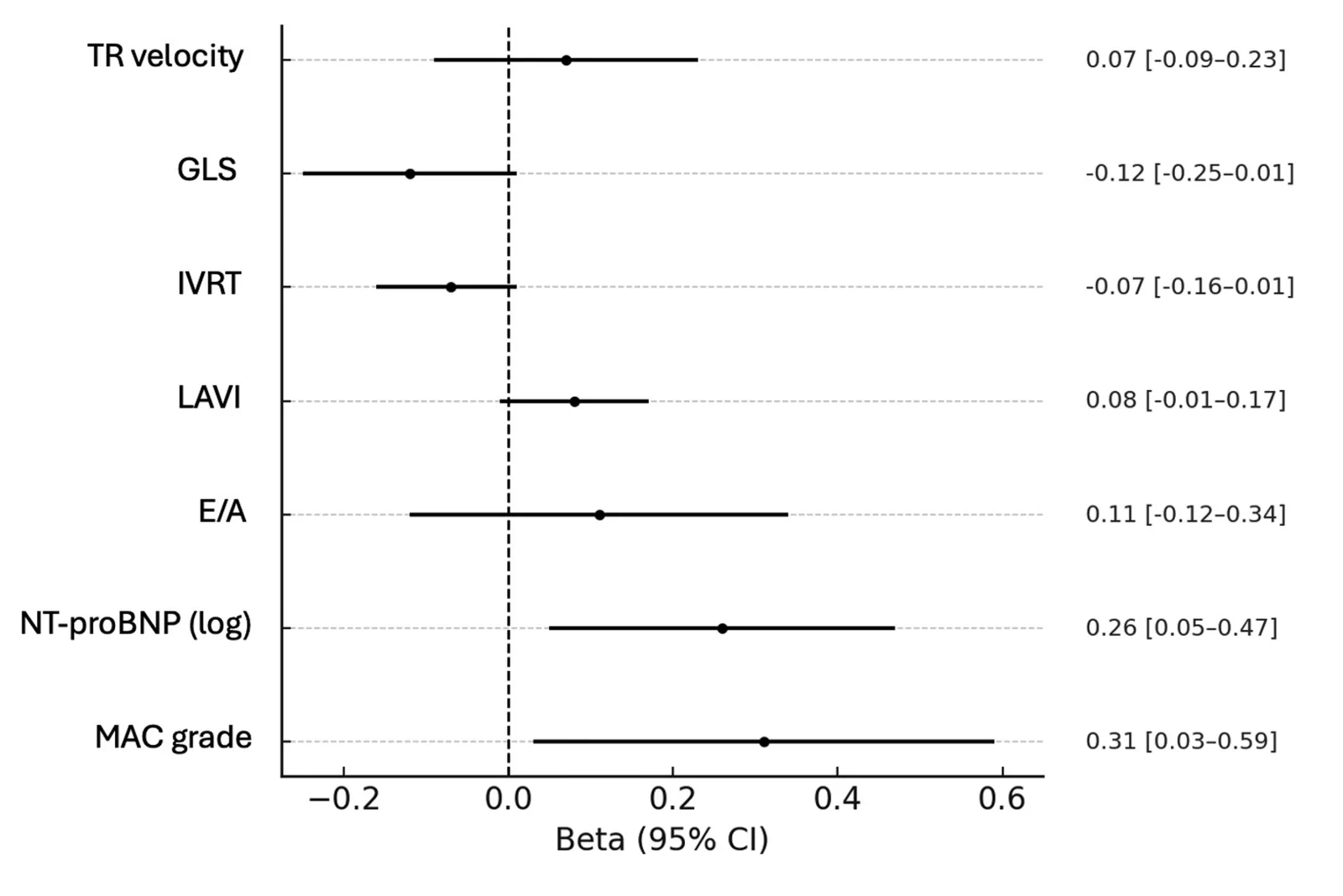
Forest plot of independent predictors of the HFA-PEFF score. CI = confidence interval, GLS = global longitudinal strain, HFA-PEFF = Heart Failure Association Pretest Assessment, Echocardiography and Natriuretic Peptide, Functional Testing, Final Etiology, IVRT = isovolumic relaxation time, LAVI = left atrial volume index, MAC = mitral annular calcification, NT-proBNP = N-terminal pro–B-type natriuretic peptide, TR = tricuspid regurgitation.

Across the full cohort, median NT-proBNP (log) was ~6.25, mean TRV ~2.53 m/s, mean LAVI ~35 mL/m^2^, mean E/A ~1.22, mean IVRT ~102 ms, and mean GLS ~−18.7%. Although these indices varied with MAC severity, their independent explanatory values were consistently smaller than those of MAC grade and NT-proBNP in the multivariable context.

## 4. Discussion

This study demonstrates a robust, graded relationship between MAC severity and multiple orthogonal indicators of HFpEF: invasive congestion (PCWP at rest and with fluid challenge), H_2_FPEF probability score, and HFA-PEFF diagnostic algorithm. Each one-increment increase in MAC grade conferred ~78% higher odds of invasive HFpEF and independently increased both H_2_FPEF and HFA-PEFF scores. These associations persisted despite adjustment for canonical diastolic markers (E/A, LAVI, TRV, and IVRT) and myocardial mechanics (GLS). Taken together, these data suggest that MAC is more than an incidental echocardiographic finding in older adults; rather, advanced MAC appears to be a structural correlate independently associated with invasively confirmed HFpEF.

Because this was an observational cross-sectional study, these findings demonstrate association and should not be interpreted as evidence that increasing MAC severity causes HFpEF or progressive elevation of filling pressures. MAC severity also should not be considered a stand-alone diagnostic criterion for HFpEF.

These findings should be interpreted within the study’s sampling and verification framework. The equal allocation of participants across MAC grades was designed to facilitate internal comparisons and does not reproduce the natural distribution of MAC severity in clinical practice. In addition, the absence of a non-MAC comparator prevents assessment of whether the observed hemodynamic profile is specific to MAC. Finally, invasive hemodynamic assessment was performed in 152 of 160 participants, whereas 8 patients with low-probability noninvasive scores did not undergo catheterization; therefore, analyses involving invasively defined HFpEF apply specifically to the catheterized subset.

MAC is a fibrocalcific degeneration of the mitral annulus linked to aging, hypertension, CKD, diabetes, altered calcium–phosphate metabolism, and systemic inflammation.^[5,14–16]^ Progressive annular calcification stiffens the atrioventricular junction, impairs annular dynamics and atrial–ventricular coupling, and can propagate into leaflets, chordae, and the LV inflow tract.^[2,5,14–16]^ These anatomical changes plausibly elevate LV filling pressures by increasing transmitral gradients even without overt stenosis, worsening annular recoil and early diastolic suction, and promoting atrial myopathy with left atrium (LA) enlargement and reduced reservoir function.^[2,5,14–16]^ The present cohort’s stepwise increase in PCWP across MAC grades, accentuated by preload augmentation, aligns with this mechanical model of impaired diastolic compliance and reserve.^[5,15,16]^

Prior evidence suggests that E/e′ may be less reliable in the setting of MAC owing to acoustic shadowing and annular rigidity, especially in moderate-to-severe calcification. As shown in the study by Abudiab et al^[17]^ and in the recent recommendations from the ASE for the evaluation of LV diastolic function and HFpEF by echocardiography,^[11]^ Doppler-derived E/A and IVRT values maintain their diagnostic accuracy in such populations, offering a more robust estimation of LV filling pressures. Based on this evidence, we chose to substitute E/A and IVRT in our regression and correlation analyses instead of E/e′, although we retained the E/e′ ratio for actual score calculations to remain consistent with the scoring system algorithms. As shown in Table 1, E/e′ differed significantly between MAC grade 1 and the higher grades, but no significant differences were observed among grades 2 to 4. In contrast, IVRT demonstrated progressive differentiation, with significant differences both between grades 1 and 2 and between lower (1–2) and higher (3–4) MAC grades. These findings suggest that in the presence of increasing annular calcification, IVRT may provide a more consistent reflection of diastolic hemodynamics than E/e′, which may be influenced by annular structural distortion.

Previous studies have linked MAC with adverse cardiac remodeling, impaired diastolic function, and elevated filling pressures.^[18–21]^ Recent investigations have further characterized the relationship between MAC and diastolic dysfunction. In one study, MAC was associated with diastolic dysfunction and adverse effects on LV performance. Participants with MAC demonstrated elevated diastolic filling pressures together with impaired LV diastolic function compared with those without MAC.^[18]^ Further research has revealed a connection between MAC and left atrial enlargement, which may contribute to the onset of diastolic dysfunction.^[19]^ Enlargement of the LA can facilitate the emergence of arrhythmias such as AF, thereby increasing the risk of HF. Additionally, MAC is associated with LV hypertrophy, a condition that exacerbates diastolic dysfunction. A specific study revealed that individuals with LV hypertrophy and concurrent MAC display more severe diastolic dysfunction.^[20]^ Consistent with these findings, our data demonstrated a progressive increase in LV mass index across ascending MAC grades, further supporting the concept that structural cardiac remodeling intensifies in parallel with the extent of annular calcification. MAC frequently coexists with other cardiovascular risk factors, including coronary artery disease and hypertension, which can also precipitate diastolic dysfunction.^[6]^

In summary, the impact of MAC on diastolic dysfunction underscores the complex interplay between various factors. A deeper understanding of this relationship may be pivotal for advancing the prevention and management of cardiovascular diseases. In our study, we demonstrated a significant correlation between MAC grade and HFpEF score, highlighting that not only the presence of MAC but also its severity is a noteworthy factor in the evaluation of patients with a diagnosis or suspicion of HFpEF.

Among demographic characteristics, age is a major risk factor for HFpEF, and 60% to 70% of patients with HFpEF are over 65 years of age.^[1]^ To reduce the potential bias stemming from the known associations between age, MAC, and HFpEF, we deliberately included patient groups with comparable age distributions. Similarly, special attention was given to achieving a balanced distribution of hypertension and DM across the study groups, as these risk factors are known to influence outcomes. The simultaneous presence of type 2 diabetes mellitus (T2DM) and HF, characterized by reduced ejection fraction (HFrEF) or preserved ejection fraction (HFpEF), is common, affecting 30% to 40% of patients.^[21]^ This coexistence is linked to an increased risk of hospitalization due to HF, as well as increased rates of all-cause and cardiovascular (CV) mortality. The key contributors to HF in individuals with T2DM include coronary artery disease, hypertension, and the direct harmful impact of T2DM on myocardial tissue. Additionally, T2DM is frequently undiagnosed in patients with HF and vice versa, highlighting the necessity for the proactive identification of both conditions in clinical settings.

Obesity, high BMI, hypertension, and AF are common risk factors associated with HFpEF. The estimated prevalence of obesity in patients with HFpEF is 30% to 40%, and obesity is associated with worse symptoms, poorer quality of life, and adverse prognosis.^[1,22]^ Among the several cardiovascular risk factors associated with HFpEF, reports from large-scale trials and clinical databases indicate that hypertension is present in 50% to 90% of patients with HFpEF.^[23]^ This prevalence is significantly higher than that reported in the general population and exceeds the rates observed in patients with HFrEF. In extensive studies on HFpEF, the incidence of AF can reach as high as 50%. Given the strong influence of these comorbidities on HFpEF pathophysiology, our study design intentionally aimed to homogenize groups with respect to BMI, hypertension, diabetes, and AF, allowing for a more accurate evaluation of the independent association between MAC severity and HFpEF-related diagnostic scores.

For identifying objective evidence of HFpEF, several echocardiographic estimates of LV filling pressures have been demonstrated, but arguably, the most studied parameters used in clinical practice to date are the transmitral flow pattern, LA volume index, mitral annular e′ velocity, E/e′ ratio, TRV, and LV mass index. The prevalence of pulmonary hypertension has been shown to vary across studies, reaching up to 55%.^[24,25]^ We found that TRV values were positively correlated with MAC grade and that these values were significantly different across the groups. Although LVEF was similar, the LAVI, E/A ratio, IVRT, GLS, and relative wall thickness values were significantly different between the groups (Table 1). In our study, the GLS values decreased from grade 1 to grade 4. Notably, grade 4 exhibited a significantly lower GLS than the other groups, indicating a deterioration in LV systolic function as the severity of MAC increased. However, there were no significant differences in GLS among the lower grades, suggesting that the impact on systolic function became pronounced only at higher levels of calcification.

Echocardiography is the first-line imaging modality for the assessment and diagnosis of MAC. Systematic assessment includes evaluation of the mitral valve annulus, leaflets, and subvalvular apparatus for the presence, location, and extent of calcification and thickening. Although previously considered a passive degenerative process, the current understanding of MAC pathogenesis is still evolving. Data from a recent nuclear imaging study suggested that patients with MAC had increased local calcific and inflammatory activity.^[26]^

In a recent study, the incidence of MAC was 14% in 13,483 consecutive patients.^[27]^ Moreover, MAC was associated with higher rates of death and positively correlated with myocardial infarction, stroke, incidental HF, AF, and composite major adverse cardiovascular events.^[28]^ These findings highlight the importance of evaluating both the presence and severity of MAC in patients with HFpEF or in those suspected of having it.

We found a stronger effect size of MAC on the HFA-PEFF score than on the H_2_FPEF score, which likely reflects algorithmic composition. HFA-PEFF incorporates structural and functional echo domains plus natriuretic peptides, which are closely tied to MAC’s mechanistic footprint, whereas H_2_FPEF gives substantial weight to clinical variables (BMI, age, antihypertensive use, and AF) and E/e′/pulmonary artery systolic pressure. The fact that MAC remained independently associated with both scores suggests that it captures the structural diastolic burden that is not fully accounted for by conventional indices.

In this study, invasive hemodynamic measurements provided an important reference for interpreting the echocardiographic findings. While MAC grade 1 and 2 patients demonstrated similar PCWP values at rest and after fluid challenge, grades 3 and 4 exhibited significantly higher filling pressures. The stepwise increase in PCWP paralleled the increasing diagnostic probability of HFpEF, with the highest rates observed in MAC grade 4. These findings demonstrate that the graded association between MAC severity and higher HFpEF scores was paralleled by progressively higher filling pressures in the catheterized subset. Importantly, differences between grades 3 and 4 underscore the progressively greater hemodynamic burden observed across increasing MAC grades.

Our results align with previous studies demonstrating the value of invasive hemodynamic assessment in patients with suspected HFpEF.^[4,29]^ These studies highlighted the importance of provocative hemodynamics in unmasking elevated filling pressures in borderline cases. In our cohort, invasive assessment enabled more robust classification of HFpEF within the catheterized subset, particularly among patients with higher MAC grades. MAC severity may reflect structural remodeling that coexists with abnormal diastolic hemodynamics; however, the cross-sectional design does not establish the direction or mechanism of this relationship.

Thus, invasive hemodynamic assessment supports the interpretation of MAC grading as an adjunctive structural feature within HFpEF evaluation. Grades 3 and 4 were associated with markedly abnormal hemodynamic profiles, but these data do not establish prognostic significance or a stand-alone diagnostic role for MAC. In the adjusted model, each 1-category increase in MAC grade was associated with 78% higher odds of meeting the invasive hemodynamic criteria for HFpEF.

In clinical practice, MAC grading should be viewed as complementary to, rather than a replacement for, established assessment of diastolic function and validated HFpEF diagnostic algorithms. HFpEF evaluation should remain based on the integrated assessment of symptoms and signs, comorbidities, natriuretic peptides, conventional and advanced echocardiographic measures, and validated tools such as the H_2_FPEF and HFA-PEFF algorithms. Within this framework, the presence of advanced MAC may provide additional structural context when the noninvasive assessment is equivocal. However, MAC severity alone neither establishes an HFpEF diagnosis nor constitutes an independent indication for invasive hemodynamic testing. Functional or invasive testing should continue to be guided by the overall clinical probability and the degree of residual diagnostic uncertainty.

A structured assessment may be particularly useful in patients with MAC and unexplained dyspnea. Initial evaluation should integrate clinical characteristics and comorbidities with natriuretic peptide concentrations, electrocardiographic findings, and a comprehensive echocardiographic assessment of diastolic function. Established H_2_FPEF and HFA-PEFF scores can then be used to organize diagnostic probability. In patients with intermediate probability or discordant findings, functional testing or invasive hemodynamic assessment may be considered according to current diagnostic pathways. Within this risk-based framework, MAC grade may serve as an additional structural feature that increases clinical awareness of possible diastolic impairment, but its incremental diagnostic value beyond established algorithms requires prospective validation.

The heterogeneous pathophysiology of HFpEF argues against reliance on any single structural or functional marker. Multimodal assessment combining clinical risk factors, biomarkers, electrocardiographic information, conventional and advanced echocardiographic measures, and validated HFpEF scores may provide a more clinically informative estimate of diastolic risk. Routinely available electrocardiographic variables may also contribute complementary information; an electrocardiographic diastolic index has, for example, demonstrated an association with echocardiographically defined diastolic dysfunction.^[30]^ Clinical and cumulative hemodynamic exposures may also modify diastolic function over time; prior work has reported an association between higher parity and impaired LV diastolic function, illustrating the broader importance of patient-specific exposures when evaluating diastolic risk.^[31]^ More broadly, contemporary prediction models increasingly integrate multiple routinely available variables to provide individualized cardiovascular risk estimates; a recent heart-rate-variability nomogram for AF illustrates this integrated prediction-model approach, although it addresses a different clinical outcome.^[32]^ Our findings suggest that MAC grade could be investigated as 1 component of such an integrated framework rather than as an independent diagnostic test.

Irrespective of MAC severity, recognition of a high-risk diastolic phenotype should prompt careful management of modifiable cardiovascular contributors in accordance with contemporary guidelines, including optimization of blood pressure, weight, and metabolic risk, and appropriate treatment of AF and ischemic heart disease when present. HFpEF-specific therapy should be considered when established diagnostic criteria are fulfilled rather than on the basis of MAC severity alone. Whether earlier recognition and treatment in patients with MAC can modify subsequent structural remodeling, MAC progression, or clinical outcomes requires prospective longitudinal investigation.

Prospective studies should test whether MAC progression parallels diastolic worsening, whether MAC integrates with emerging HFpEF phenomapping, and whether targeted therapies (e.g., mineral metabolism modulation and anti-inflammatory strategies) alter both MAC trajectory and HFpEF outcomes. Future work should explore automated, quantitative MAC burden (computed tomography calcium scoring or echo-based radiomics) and its incremental value over current algorithms.

Contemporary HFpEF imaging increasingly incorporates left atrial strain and right ventricular strain metrics, which provide insight into atrial myopathy and right ventricle-pulmonary coupling. Although these advanced strain parameters were not available in our dataset, MAC grading may represent a complementary structural marker reflecting chronic atrioventricular remodeling. Future studies integrating MAC burden with LA and right ventricle strain may further refine phenotypic stratification.

From a clinical standpoint, when advanced MAC coexists with unexplained dyspnea and an otherwise equivocal noninvasive assessment, it may contribute to the overall clinical suspicion of HFpEF. Decisions regarding functional or invasive hemodynamic testing should remain based on the integrated clinical probability.

Several limitations should be considered. First, the cross-sectional observational design establishes association but cannot determine causality, temporal sequence, or prognostic significance. Second, the stratified enrollment strategy was designed to provide balanced MAC-grade groups for internal comparisons and therefore does not reproduce the natural distribution of MAC severity in an unselected population, limiting external generalizability. Third, because all participants had echocardiographically confirmed MAC, the absence of a non-MAC comparator prevents assessment of the specificity of these findings for MAC. Fourth, RHC was performed in 152 of 160 participants; the 8 patients with low-probability noninvasive scores were not catheterized, and invasive analyses therefore apply only to the catheterized subset and may be subject to verification bias. Fifth, exercise RHC was not available, and rapid fluid challenge was used as an alternative provocative maneuver. Finally, left atrial and right ventricular strain were not assessed. Future studies using unselected cohorts, non-MAC comparator groups, comprehensive strain imaging, and longitudinal follow-up are required to determine whether MAC severity provides incremental diagnostic or prognostic information beyond established HFpEF algorithms.

## 5. Conclusion

Increasing MAC severity was associated with progressively greater diastolic and hemodynamic abnormalities and with a higher prevalence of invasively defined HFpEF in the catheterized subset. MAC grade was also independently associated with H_2_FPEF and HFA-PEFF scores. These findings support considering MAC severity as a complementary structural feature within an integrated HFpEF assessment rather than as a stand-alone diagnostic marker. Prospective studies in unselected populations, including non-MAC comparators, are needed to determine whether MAC grading provides incremental diagnostic or prognostic value beyond established clinical, biomarker, echocardiographic, and HFpEF scoring approaches.

## Acknowledgments

We would like to express our special thanks to Associate Professor Ali Kemal Cabuk for his contributions to our study.

## Author contributions

**Conceptualization:** Gizem Cabuk.

**Data curation:** Gizem Cabuk, Burcu Uludag Artun.

**Formal analysis:** Gizem Cabuk, Burcu Uludag Artun.

**Investigation:** Gizem Cabuk, Burcu Uludag Artun.

**Methodology:** Gizem Cabuk.

**Project administration:** Burcu Uludag Artun.

**Resources:** Gizem Cabuk, Burcu Uludag Artun.

**Software:** Gizem Cabuk, Burcu Uludag Artun.

**Supervision:** Gizem Cabuk.

**Validation:** Gizem Cabuk.

**Visualization:** Gizem Cabuk, Burcu Uludag Artun.

**Writing – original draft:** Gizem Cabuk.

**Writing – review & editing:** Gizem Cabuk, Burcu Uludag Artun.
